# The Roles of Incretin Hormones GIP and GLP-1 in Metabolic and Cardiovascular Health: A Comprehensive Review

**DOI:** 10.3390/ijms27010027

**Published:** 2025-12-19

**Authors:** Dai Yamanouchi

**Affiliations:** 1 Division of Vascular Surgery, Department of Surgery, University of Wisconsin School of Medicine and Public Health, Madison, WI 53705, USA; yamano@surgery.wisc.edu or dai.yamanouchi@fujita-hu.ac.jp; 2 Department of Vascular Surgery, Fujita Health University, Toyoake 470-1192, Japan

**Keywords:** incretin hormones, GLP-1 receptor agonists, GIP signaling, tirzepatide, dual agonists, insulin secretion, atherosclerosis, cardiovascular protection, metabolic disease, obesity treatment

## Abstract

The incretin hormones glucose-dependent insulinotropic polypeptide (GIP) and glucagon-like peptide-1 (GLP-1) play central roles in metabolic and cardiovascular regulation. GLP-1 receptor agonists (GLP-1RAs) are established therapies for type 2 diabetes mellitus (T2DM) and obesity because of their insulinotropic effects, weight reduction, and proven cardiovascular benefit in trial level. In contrast, GIP was historically overlooked due to reduced β-cell responsiveness in T2DM. The development of dual GIP/GLP-1 receptor agonists has reshaped this view. Tirzepatide, the first-in-class co-agonist, an antidiabetic medication to treat type 2 diabetes and for weight loss, provides superior glycemic control and weight loss compared with selective GLP-1RAs in clinical trials, demonstrating synergistic actions between the two incretin pathways. This review summarizes key physiology, pathophysiology, and therapeutic evidence in incretin biology. We describe secretion patterns, receptor distributions, and distinct actions of GIP and GLP-1, as well as alterations in incretin signaling in T2DM and obesity. Cardiovascular protective mechanisms are outlined, including improvements in lipid metabolism, reductions in blood pressure, enhanced endothelial nitric oxide activity, suppression of macrophage inflammation, decreased foam-cell formation, and stabilization of atherosclerotic plaques. At the therapeutic level, emerging directions—such as dual and triple agonists—and unresolved questions regarding long-term vascular effects of GIP and the potential for genotype-guided incretin therapy are also discussed. Collectively, these findings highlight an emerging shift toward integrated incretin-axis modulation as a therapeutic strategy for metabolic and cardiovascular disease.

## 1. Introduction

### 1.1. The Evolving Story of Incretins

Incretins are gut-derived peptide hormones secreted in response to nutrient intake that enhance glucose-dependent insulin secretion and thereby reduce postprandial blood glucose levels. The two principal incretin hormones in humans are glucose-dependent insulinotropic polypeptide (GIP) and glucagon-like peptide-1 (GLP-1). The term incretin effect refers to the phenomenon whereby oral glucose administration elicits a greater insulin response than intravenous glucose delivery, reflecting the physiological contribution of incretin hormones to postprandial glucose homeostasis. Incretin biology has become increasingly central to modern metabolic therapeutics, reshaping not only how glucose regulation is understood but also how obesity, cardiovascular disease, and multisystem metabolic dysfunction are treated. The two primary incretin hormones—glucose-dependent insulinotropic polypeptide (GIP) and glucagon-like peptide-1 (GLP-1)—are secreted from enteroendocrine cells of the small intestine following nutrient ingestion. Together, they orchestrate the “incretin effect, a fundamental physiological mechanism that amplifies glucose-stimulated insulin secretion after oral glucose intake compared with intravenous administration [1,2]. This effect accounts for roughly 50–70% of the total postprandial insulin release in healthy individuals and has served as a cornerstone for understanding gut–pancreas communication in energy and glucose homeostasis. In this review, we use the term incretin axis to denote the integrated network through which GIP and GLP-1 coordinate metabolic and cardiovascular regulation, including their seemingly biphasic and sometimes paradoxical effects in health and disease. The term twincretins refers to unimolecular dual agonists that simultaneously activate both the GIP and GLP-1 receptors.

For decades, research and drug development overwhelmingly focused on GLP-1. Its ability to stimulate insulin secretion was found to be largely preserved in patients with type 2 diabetes (T2DM), providing a clear rationale for its development as a major therapeutic target [3,4]. Beyond its glucose-dependent enhancement of insulin secretion, GLP-1 was shown to suppress inappropriate glucagon release, slow gastric emptying, promote satiety through central appetite pathways, and favor weight reduction—properties that aligned seamlessly with clinical goals for diabetes and obesity management. This led to a highly successful class of drugs—GLP-1 receptor agonists (GLP-1RAs)—that established a new benchmark for metabolic therapy, creating a high bar for any subsequent innovations [5]. The clinical success of GLP-1RAs also broadened the scope of incretin biology, revealing important cardioprotective effects, bone remodeling, and skeletal muscle biology that extended their relevance far beyond glycemic control [1,2,6,7].

In contrast, GIP was historically relegated to a secondary role, often considered the “neglected incretin” [1]. Although initially identified as a potent stimulator of glucose-dependent insulin secretion, its action was found to be markedly diminished in patients with T2DM, leading to the assumption that GIP was rendered functionally irrelevant in the diabetic state [3]. This apparent “GIP resistance” resulted in the hormone being largely sidelined from therapeutic consideration for many years. Early clinical studies consistently showed blunted β-cell responsiveness to GIP in T2DM, reinforcing the belief that the hormone lacked translational potential and was unlikely to contribute meaningfully to metabolic therapy [8]. However, this long-held dogma has been dramatically overturned by the development of dual GIP/GLP-1 receptor co-agonists, most notably tirzepatide. Clinical trials have demonstrated that tirzepatide achieves superior reductions in both glycated hemoglobin (HbA1c) and body weight compared to even the most potent selective GLP-1RAs [9,10]. This remarkable efficacy has not only reignited scientific interest in GIP but has also revealed a more complex, synergistic relationship between these two hormones, prompting a fundamental re-evaluation of their integrated roles in metabolic regulation [1].

This review synthesizes the current understanding of these two pivotal hormones, beginning with their foundational physiology.

### 1.2. Fundamental Physiology of the Incretin System

A thorough understanding of the fundamental physiology of GIP and GLP-1 is essential to fully appreciate their roles in metabolic homeostasis and their therapeutic manipulation in disease. Although both hormones participate in the incretin response, they arise from distinct enteroendocrine cell populations, are secreted under different nutritional contexts, and engage separate receptors with unique tissue distributions. These differences shape their individual physiological actions and also determine how they interact when both pathways are stimulated simultaneously.

GIP, secreted primarily from K cells in the proximal small intestine, responds robustly to dietary fats and carbohydrates, while GLP-1, derived from L cells predominantly located in the distal ileum and colon, is released following nutrient transit through the gut [11,12]. Their receptors are widely distributed across pancreatic islets, adipose tissue, the gastrointestinal tract, the cardiovascular system, bone, and the central nervous system, allowing each hormone to influence a broad array of metabolic processes beyond insulin secretion [11,12].

Together, these characteristics form the biological foundation for their complementary roles in glucose regulation, appetite and energy balance, lipid handling, and cardiovascular function. Understanding these physiological principles is critical not only for interpreting normal metabolic regulation but also for understanding why pharmacologic targeting of these pathways—individually or in combination—has produced such profound therapeutic effects. The following sections provide a detailed examination of these core physiological mechanisms to establish the groundwork for later discussion of their roles in disease modulation and therapeutic innovation.

## 2. Physiology of GIP and GLP-1

### 2.1. Secretion and Metabolism

GIP originates from enteroendocrine K-cells predominantly situated in the duodenum and jejunum, whereas GLP-1 is mainly released from L-cells, which become more abundant toward the ileum and colon [13,14,15]. Their release is triggered primarily by the ingestion and absorption of nutrients, particularly carbohydrates and fats, and to a lesser extent, proteins [1,16]. Following secretion into the bloodstream, both hormones are characterized by extremely short half-lives. Natural GLP-1, for example, has a half-life of only about two minutes [17]. This rapid clearance is due to enzymatic degradation by dipeptidyl peptidase-IV (DPP-IV), an enzyme widely distributed throughout the body that efficiently inactivates both hormones [2,17].

### 2.2. Receptor Distribution

The biological effects of GIP and GLP-1 are mediated through their actions as agonists at specific G protein-coupled receptors, the GIP receptor (GIPR) and GLP-1 receptor (GLP-1R). The widespread distribution of these receptors throughout the body accounts for the pleiotropic effects of these hormones, extending far beyond their primary role in glucose regulation. The table below (Table 1) synthesizes their presence in key tissues relevant to metabolic and cardiovascular health.

This broad receptor distribution is strategically significant. It underlies the capacity of incretin hormones to influence a network of organ systems, including the pancreas, heart, vasculature, and central nervous system. The presence of both GIP and GLP-1 receptors in the heart, blood vessels, and adipose tissue provides a direct anatomical and molecular basis for the cardiovascular benefits observed in clinical trials, suggesting these effects are not solely secondary to metabolic improvements but may also involve direct tissue-level signaling [7].

### 2.3. Core Biological Actions

While both GIP and GLP-1 share the primary function of stimulating insulin secretion, they possess distinct and sometimes opposing actions, particularly concerning glucagon secretion and gastrointestinal function.

#### 2.3.1. Regulation of Glucose Homeostasis

The defining action of both GIP and GLP-1 is their ability to augment glucose-stimulated insulin secretion from pancreatic β-cells, a process that forms the cornerstone of the incretin effect [18]. However, their influence on the counter-regulatory hormone glucagon differs significantly [1]:

GLP-1 potently suppresses glucagon secretion from pancreatic α-cells when glucose levels are elevated, contributing to its glucose-lowering effect [19].

GIP, in contrast, can stimulate glucagon secretion, particularly when glucose levels are low or normal [20,21].

This functional divergence highlights their complementary roles. In healthy individuals, GIP is considered the dominant physiological incretin hormone, contributing more substantially to the total post-meal insulin response than GLP-1 [1,21,22].

#### 2.3.2. Gastrointestinal and Appetite Regulation

GLP-1 and GIP also exert markedly different effects on the gastrointestinal tract and central regulation of appetite.

GLP-1 significantly slows gastric emptying, which retards the delivery of nutrients into circulation and helps blunt postprandial glucose excursions [23,24]. At pharmacological concentrations, it also acts on the central nervous system to reduce appetite, decrease food intake, and promote satiety, which are key mechanisms underlying its efficacy in weight management [5].

GIP has no effect on gastric emptying [25]. While animal studies suggest a potential role for GIP in appetite regulation, this has not been confirmed in human studies, where its effects on food intake remain unsubstantiated [1,26].

Having established their functions in a healthy physiological state, it is crucial to examine how their roles are altered in the context of metabolic disease.

## 3. Pathophysiological Role in Type 2 Diabetes and Obesity

The therapeutic utility of incretin-based drugs stems directly from the altered function and response of the incretin system in metabolic diseases. The key pathophysiological changes observed in T2DM and obesity explain why GLP-1 became a successful drug target while GIP was initially overlooked.

### 3.1. The Diminished Incretin Effect in T2DM: The “GIP Resistance” Phenomenon

A hallmark of T2DM is a significantly reduced incretin effect, meaning that oral glucose fails to elicit the robust insulin response seen in healthy individuals [27]. While GLP-1 secretion in T2DM is largely preserved or slightly diminished, reports on GIP secretion are inconsistent, with studies showing responses ranging from normal to somewhat increased [1]. The primary driver of this dysfunction is a profound blunting of the insulinotropic action of GIP [1,28]. This phenomenon, often referred to as “GIP resistance,” means that even high concentrations of GIP fail to effectively stimulate insulin secretion from the diabetic pancreas [3,29].

In stark contrast, the ability of GLP-1 to stimulate insulin secretion is largely preserved in individuals with T2DM [3,4]. This crucial difference provides the foundational rationale for the development of GLP-1RAs as a highly effective therapeutic class; they effectively “replace” the lost incretin effect by activating a signaling pathway that remains functional in the disease state. The precise molecular mechanisms underlying this GIP resistance, and whether this state can be pharmacologically reversed to restore GIP’s insulinotropic efficacy, remain areas of intense clinical investigation.

### 3.2. Impact on Adipose Tissue and Lipid Metabolism

Beyond glucose control, incretin hormones play a role in lipid metabolism and adipose tissue function, where their effects diverge.

GIP has an anabolic role in white adipose tissue. It promotes the clearance of triglycerides from the circulation and their subsequent storage in adipocytes by enhancing the activity of lipoprotein lipase [1,30,31,32,33]. This function has fueled a long-standing debate about whether GIP may have a potentially “obesogenic” role by promoting fat deposition [34,35]. Crucially, while GIP’s insulinotropic effect is blunted in T2DM, its actions on adipose tissue may be preserved, potentially contributing to altered lipid partitioning and fueling the debate over its role in the pathophysiology of metabolic disease.

GLP-1RAs, conversely, are associated with improvements in the overall lipid profile. Clinical studies have shown that they consistently reduce fasting total cholesterol, triglycerides (TGs), and LDL-cholesterol while moderately increasing HDL-cholesterol [36].

These differing roles in metabolic disease set the stage for understanding their equally important and largely beneficial implications for cardiovascular health.

## 4. Cardiovascular Implications and Anti-Atherosclerotic Effects

Beyond their established benefits for glycemic and weight control, a major advantage of incretin-based therapies—particularly GLP-1RAs—is their proven ability to provide cardiovascular protection (Table 2). The mechanisms responsible for this benefit are multifaceted, involving both indirect improvements in systemic risk factors and direct, vasoprotective effects on the arterial wall, as extensively reviewed by Wang et al. [7].

### 4.1. Clinical Evidence from Cardiovascular Outcome Trials (CVOTs)

Large-scale, long-term cardiovascular outcome trials (CVOTs) have provided definitive evidence that several GLP-1RAs reduce the risk of major adverse cardiac events (MACEs) in patients with T2DM [7]. Across multiple outcome trials, GLP-1RAs consistently lowered major adverse cardiovascular events. Liraglutide improved cardiovascular outcomes in LEADER, semaglutide showed significant stroke and MI reductions in SUSTAIN-6, and dulaglutide demonstrated similar benefits in REWIND.

LEADER Trial (*n* ≈ 9300; median follow-up 3.8 years): Liraglutide demonstrated a 13% reduction in the risk of MACE (a composite of cardiovascular death, nonfatal myocardial infarction, and nonfatal stroke) and a significant 22% reduction in cardiovascular death compared to placebo [7,37].

SUSTAIN-6 Trial (*n* ≈ 3300; median follow-up 2.1 years): Semaglutide led to a 26% risk reduction in the primary composite MACE outcome, driven by a notable decrease in nonfatal stroke and nonfatal myocardial infarction [7,38].

REWIND Trial (*n* ≈ 9900; median follow-up 5.4 years): Dulaglutide showed a significant reduction in MACE, an effect largely driven by a 24% reduction in nonfatal stroke [7,39].

### 4.2. Indirect Cardioprotective Mechanisms: Modulating Systemic Risk Factors

A substantial portion of the cardiovascular benefit derived from incretin-based therapies stems from their positive impact on systemic cardiometabolic risk factors.

Improvement in Dyslipidemia: As noted previously, GLP-1RAs and dual GIP/GLP-1RAs effectively improve lipid profiles by reducing levels of triglycerides and LDL-cholesterol while raising HDL-cholesterol, which helps mitigate a key driver of atherosclerosis [7,40]. This improvement extends to postprandial lipemia, where GLP-1RAs have been shown to blunt the rise in triglycerides and atherogenic chylomicron remnants, a key contributor to cardiovascular risk that is often overlooked in fasting lipid panels [41].

Reduction in Hypertension: Clinical trials have consistently shown that GLP-1RAs and the dual agonist tirzepatide significantly reduce systolic blood pressure (SBP) [42,43]. The underlying mechanisms are thought to involve direct renal effects that promote urinary sodium excretion (natriuresis) as well as vasodilation of blood vessels [44,45,46,47].

### 4.3. Direct Anti-Atherosclerotic Mechanisms

In addition to improving systemic risk factors, incretins exert direct protective effects on the vasculature that are independent of their metabolic actions. These cellular mechanisms, synthesized from recent reviews [7], directly counteract the key processes involved in the formation and progression of atherosclerotic plaques. In Table 3, direct vascular and anti-atherosclerotic mechanisms of GLP-1RA and GIP receptor agonist (GIP-RA) are summarized.

#### 4.3.1. Preserving Endothelial Function

Incretin receptor agonists support vascular health by promoting endothelial nitric oxide synthesis, limiting inflammatory signaling, reducing lipid uptake by macrophages, and curbing maladaptive smooth-muscle proliferation—mechanisms that collectively contribute to plaque stability.

Promoting Vasodilation: GLP-1 RAs enhance the production of nitric oxide (NO), a potent vasodilator, by activating the enzyme endothelial nitric oxide synthase (eNOS) [48,49].

Reducing Inflammation and Oxidative Stress: GLP-1 RAs inhibit pro-inflammatory signaling pathways like NF-κB, thereby reducing the expression of adhesion molecules that recruit inflammatory cells to the vessel wall [50,63].

Decreasing Permeability: GLP-1RAs strengthen the endothelial barrier, reducing the infiltration of lipoproteins and inflammatory cells, and inhibit endothelial cell apoptosis [51,52].

#### 4.3.2. Modulating Macrophage Activity and Plaque Inflammation

IRAs intervene at critical steps of plaque development by:

Inhibiting Foam Cell Formation: GLP-1 RAs reduce the formation of macrophage-derived foam cells—a hallmark of early atherosclerotic lesions—by inhibiting the uptake of oxidized LDL (ox-LDL) and up-regulating cholesterol efflux transporters like ABCA1 that remove excess cholesterol from cells [54,55,56,64].

Attenuating Plaque Inflammation: GLP-1R activation promotes the polarization of macrophages toward an anti-inflammatory M2 phenotype, which is associated with tissue repair, rather than the pro-inflammatory M1 phenotype that drives plaque progression [53,58].

#### 4.3.3. Stabilizing Vascular Smooth Muscle Cells (VSMCs)

GLP-1RAs contribute to the stability of advanced atherosclerotic plaques, making them less prone to rupture, by:

Suppressing Abnormal VSMC Behavior: GLP-1RAsinhibit the abnormal proliferation, migration, and phenotypic switching of VSMCs from a stable contractile state to an unstable synthetic state [57,60].

Strengthening the Fibrous Cap: GLP-1R activation increases the collagen content within the plaque’s protective fibrous cap while reducing the expression of matrix metalloproteinases (MMPs), which are enzymes that degrade the cap and increase rupture risk [59,62].

This deep understanding of incretin biology has paved the way for an evolution in therapeutic strategies, from targeting single receptors to harnessing dual-hormone action.

### 4.4. Dual Agonists Effect

A nuanced understanding of the dual agonists requires examining the individual contributions of GIP and GLP-1 to these vascular effects. While GIP is known to regulate the physiology of blood vessels, such as controlling blood flow rate in the hepatic portal veins [61], evidence suggests that the direct cardioprotective action on endothelial cells is primarily mediated by GLP-1 [65]. One study suggested that GLP-1, but not GIP, provides protective effects on endothelial function linked to increased levels of the second messenger, cyclic adenosine monophosphate (cAMP) [61]. Therefore, the immediate, anti-atherosclerotic benefits reported in short-term studies are largely driven by the GLP-1 component. GIP’s major contribution to superior clinical outcomes in dual agonists is consequently mediated more through its powerful systemic effects, specifically the enhanced management of total body and ectopic fat, which reduces the overall burden on the cardiovascular system. Direct vascular data for GIP remain limited, and the majority of robust cardiovascular outcome evidence currently derives from trials of selective GLP-1 receptor agonists. For dual GIP/GLP-1 receptor agonists, particularly in non-diabetic populations, large cardiovascular outcome trials are ongoing, and conclusions regarding direct cardiovascular benefit should therefore be considered preliminary.

## 5. The Therapeutic Landscape: From GLP-1RAs to Dual and Triple Agonists

The deepening understanding of incretin pathophysiology has directly fueled an evolution in drug development. This journey has progressed from leveraging the benefits of a single hormone to unlocking the superior potential of co-agonism, fundamentally changing the standards of care for T2DM and obesity. The advent of unimolecular dual GIP/GLP-1 receptor agonists, often termed “twincretins,” marks a significant advancement by leveraging the synergistic potential of both incretin pathways. Tirzepatide, a prominent example of this class, activates both the GLP-1 and GIP receptors simultaneously, leading to enhanced metabolic effectiveness [66]. The U.S. Food and Drug Administration (FDA) recognizes Tirzepatide (Zepbound) as a first-in-class medication for weight loss, demonstrating its unique position in the therapeutic landscape.

### 5.1. The Established Role of GLP-1 Receptor Agonists

For over a decade, GLP-1RAs have become a cornerstone in the management of T2DM and obesity. Their success is built on a solid foundation: a preserved insulinotropic effect in T2DM, robust glucose-lowering efficacy, significant weight reduction benefits, and proven cardiovascular protection in large-scale clinical trials [67,68,69]. Their established profile has made them a preferred choice for patients with T2DM and co-existing cardiovascular disease or obesity.

### 5.2. The Re-Emergence of GIP: Synergies in Dual GIP/GLP-1 Receptor Agonism

The therapeutic landscape was transformed by the arrival of tirzepatide, the first-in-class dual GIP/GLP-1 receptor agonist. Clinical trial data have shown that tirzepatide achieves unprecedented efficacy, with average HbA1c reductions of approximately 2% and body weight reductions often exceeding 10 kg—results that are superior to those seen with even the most potent selective GLP-1RAs [9,10,40].

These powerful findings have challenged the long-held view that GIP possesses no therapeutic value in T2DM [1]. The superior outcomes strongly suggest an additive or synergistic interaction between GIP and GLP-1 receptor signaling pathways [70,71]. This has sparked a new wave of research aimed at elucidating the precise mechanisms by which GIP agonism, in the context of GLP-1 receptor activation, overcomes the apparent GIP resistance seen in T2DM to produce these enhanced metabolic benefits.

## 6. Clinical Evidence for GLP-1 RAs in Non-Diabetic Patients (Secondary Prevention)

### 6.1. The SELECT Trial: The Paradigm Shift

The SELECT trial provided the definitive, robust evidence for the cardiovascular benefits of GLP-1 RAs in non-diabetic patients. This randomized controlled trial evaluated Semaglutide versus placebo over an average follow-up of nearly 40 months in 17,604 non-diabetic patients (aged ≥45 years) who had established Atherosclerotic Cardiovascular Disease (ASCVD) and a BMI ≥ 27 kg/m^2^ [72].

The primary outcome, MACE—a composite of cardiovascular death, nonfatal myocardial infarction (MI), or nonfatal stroke —was significantly reduced by Semaglutide [72]. The trial established that Semaglutide reduced the risk of MACE by 20% among non-diabetic patients with obesity and pre-existing CVD [65,73]. This benefit confirmed that the mechanism of action extends beyond blood sugar regulation, representing a potential paradigm shift in cardiovascular risk management [73].

### 6.2. Subgroup Analysis: Efficacy in Highest-Risk Cohorts

The MACE reduction benefit demonstrated in SELECT was remarkably consistent across the entire patient population, regardless of their history of established CVD [74]. A detailed prespecified analysis focused on patients with a history of Coronary Artery Bypass Grafting (CABG), a marker for high-risk, severe ASCVD [72].

Patients with prior CABG were found to have inherently higher baseline risks for MACE and all-cause mortality. However, Semaglutide consistently reduced the MACE risk compared with placebo in both patients with a CABG history (Hazard Ratio: 0.72; 95% Confidence Interval [CI]: 0.54–0.95) and those without (HR: 0.82; 95% CI: 0.73–0.92). A critical finding for clinical implementation lies in the Absolute Risk Reduction (ARR). While the relative benefit was consistent, the ARR with Semaglutide versus placebo over the approximately 3.5-year follow-up was numerically greater in patients with a history of CABG (2.3%) than in those without (1.0%). This differential ARR demonstrates that in patients with the highest pre-existing risk of recurrent events, the intervention yields the most substantial absolute clinical reward, providing a clear rationale for prioritizing this therapy in the most vulnerable non-diabetic ASCVD patients.

### 6.3. Impact on Secondary CV Outcomes and Mortality

Beyond the primary MACE endpoint, Semaglutide demonstrated consistent reductions in secondary outcomes across the SELECT cohort, lowering the risks of cardiovascular death and all-cause mortality regardless of CABG status.

Further analyses on the broader class of GLP-1 RAs confirm significant benefits. These agents reduced all-cause mortality (RR 0.88), cardiovascular death (RR 0.88), and the incidence of stroke (RR 0.86). Importantly, the magnitude of MACE and mortality effects in overweight/obese patients without diabetes was similar to the effects seen in patients with diabetes, highlighting the weight-independent nature of some protective pathways [75].

The risk of hospitalization for heart failure was notably reduced by 10% overall (RR 0.90) in GLP-1 RA trials [75]. This benefit was statistically significant and particularly pronounced in the subgroup of overweight/obese non-diabetic patients (RR 0.73; 95% CI: 0.56–0.95), reinforcing the specific utility of this class for heart failure prevention in obesity [75]. Furthermore, GLP-1 RAs were associated with a 22% reduction in the incidence of coronary revascularization (RR 0.78; 95% CI: 0.69 to 0.87) compared with placebo in overweight/obese patients without diabetes [76].

### 6.4. Awaiting Definitive Evidence: The SURMOUNT-MMO Trial

To provide definitive, prospective evidence regarding the clinical benefits of dual agonism in non-diabetic populations, the global, randomized SURMOUNT-MMO trial (estimated completion 2027) is currently underway [77].

This pivotal trial is specifically designed to investigate the impact of once-weekly Tirzepatide versus placebo on morbidity and mortality in adults with obesity or overweight, without diabetes, who are either at risk of or have established cardiovascular disease. Its scope is unique in that it assesses both primary and secondary cardiovascular disease prevention within the same outcome trial for an incretin medication in a non-diabetic population. The comprehensive primary endpoint includes a five-component composite outcome: nonfatal myocardial infarction, nonfatal stroke, coronary revascularization, heart failure events, or death from any cause. The results of SURMOUNT-MMO are necessary to fully integrate dual agonists into cardiovascular guidelines for non-diabetic individuals.

## 7. GLP-1 and GIP Influence on Bone Health and Remodeling

GLP-1 and GIP play important roles in skeletal homeostasis and bone remodeling. Evidence from GLP-1R and GIPR knockout mouse models demonstrates deterioration of bone microarchitecture and increased skeletal fragility, indicating that both incretin pathways contribute to maintaining bone health [2].

### 7.1. Bone Resorption and Formation

Both GLP-1 and GIP support bone formation and suppress bone resorption [6,7]. GIP appears to exert the more potent anti-resorptive effect, accounting for approximately 22–25% of the postprandial reduction in bone resorption in healthy adults [6,78]. Mechanistically, GIP acts directly on human osteoclasts to inhibit bone resorption, reduce osteoclast survival, and delay actin ring formation [79]. GIP also acutely stimulates bone formation and rapidly uncouples bone resorption from formation [78].

GLP-1RAs, similarly, promote bone formation through several intracellular mechanisms. They enhance osteogenic differentiation of bone marrow stromal cells (BMSCs) via the PKA/β-catenin signaling pathway [80,81], and may also activate cAMP/PKA and MAPK signaling in mature osteoblasts, further contributing to anabolic bone effects [80].

### 7.2. Bone Material Properties

Beyond cellular activity, GIP influences the intrinsic material properties of bone. Experimental studies show that GIP improves collagen maturity and reduces collagen fibril diameter in osteoblast cultures [80]. Novel multi-agonist approaches also support bone quality: for example, the dual GIP/GLP-2 analog GL-0001 enhances bone tissue material properties and increases biomechanical strength in preclinical studies [82].

### 7.3. Fracture Risk and Osteoarthritis

GLP-1RAs are considered safe with respect to fracture risk and do not increase skeletal events in patients with T2DM [83,84]. Some meta-analyses report significant reductions in fracture incidence with GLP-1RA therapy compared with other antidiabetic treatments over 52 weeks [83,85,86]. Clinically, low circulating GLP-1 levels correlate with osteoporosis, suggesting a physiological link between GLP-1 deficiency and impaired bone health [83].

GLP-1RAs also show promise in musculoskeletal disorders beyond osteoporosis. Their weight-reducing, anti-inflammatory, and immunomodulatory effects support potential therapeutic roles in osteoarthritis (OA) [87].

### 7.4. Effect of GIP on Sarcopenia

Sarcopenia is defined as the progressive loss of skeletal muscle mass and function and represents a clinically relevant concern in aging populations and in individuals undergoing significant weight loss. When sarcopenia coexists with excess adiposity, the condition is referred to as sarcopenic obesity, a phenotype associated with increased cardiometabolic risk and adverse functional outcomes. However, GIP has non-negligible side effects on muscle cells, and GIPR antagonism could be used as a new treatment for sarcopenia. In a study of GIP receptor-knockout mice, researchers discovered that GIP drives the differentiation of fibroadipogenic muscle precursors into adipocytes, which is thought to be the pathological cause of sarcopenia. GIPR antagonism functions to reduce intramuscular adipose tissue and regenerate new muscle [88]. Preclinical studies suggest that GIP signaling may influence muscle–fat lineage allocation, raising the hypothesis that GIP receptor modulation could affect skeletal muscle composition. However, current evidence is largely derived from animal and mechanistic models, and the effects of GLP-1/GIP co-agonists on muscle mass and function in humans remain to be determined in dedicated clinical studies. For the drug development industry, developing drugs to prevent muscle wasting and collaborating with the successful obesity management drug GLP-1/GIP agonists is very promising [89].

Conversely, the effects of GIP on muscle tissue are complex. GIP may promote adipogenic differentiation of muscle precursor cells, contributing to sarcopenia; thus, GIPR antagonism has been proposed as a potential therapeutic approach for sarcopenia management [88]. These findings derive primarily from preclinical and mechanistic studies, and their relevance to human physiology remains to be established. Potential effects of GIP modulation on skeletal muscle mass should therefore be viewed as a signal warranting careful monitoring in clinical studies, rather than as a confirmed adverse outcome.

## 8. Conclusions and Future Directions

Insights into incretin physiology have shifted the therapeutic landscape from single-receptor approaches to multi-agonist strategies that integrate metabolic and cardiovascular regulation. While GLP-1 has proven to be a critical pharmacological tool, with its actions on gastric emptying, appetite suppression, and a preserved insulinotropic effect in T2DM forming the basis of a highly successful class of therapeutics, GIP stands as the dominant physiological incretin in healthy individuals. The success of GIP/GLP-1 receptor co-agonists has reconciled this dichotomy, confirming that harnessing the synergy between these two pathways can unlock a new therapeutic frontier with metabolic benefits that surpass what can be achieved by targeting GLP-1 alone. Future research priorities include defining the long-term cardiovascular effects of dual and triple incretin agonists, clarifying the tissue-specific actions of GIP in humans, and identifying biomarkers or genetic determinants that may guide personalized incretin-based therapy.

## Figures and Tables

**Table 1:** Expression of GLP-1R and GIPR.

Tissue/Organ	GLP-1R	GIPR
Endocrine Pancreas	β-cells: +++	β-cells: +++
	α-cells: −/+	α-cells: ++
Heart	+ (All four chambers, particularly the sinoatrial node)	+ (All four chambers)
Blood Vessels	+ (Endothelial cells)	+ (Endothelial cells)
Adipose Tissue	+ (P in vascular cells; debated on adipocytes)	++
Bone	−/+ (Absent in cultured osteoblasts but present in bone marrow stromal cells)	++ (Present in osteoblasts and osteocytes)
Brain	++ (Present in key areas for appetite regulation like the hypothalamus and brainstem)	+ (Present in various regions including hippocampus and cortex)

Symbol definitions: +++ = abundant expression; ++ = moderate expression; + = low expression; −/+ = variable or limited expression.

**Table 2:** Major cardiovascular outcome trials of GLP-1 receptor agonists.

Trial	Agent	Population	*n*	Follow-Up	Key Results
LEADER	liraglutide	T2DM + established CVD	9340	3.8 yrs	13% ↓ MACE; 22% ↓ CV death
SUSTAIN-6	semaglutide	T2DM, high CV risk	3297	2.1 yrs	26% ↓ MACE; ↓ stroke, MI
REWIND	dulaglutide	T2DM (mostly primary prevention)	9901	5.4 yrs	12% ↓ MACE; ↓ stroke
SELECT	semaglutide	non-diabetic, obesity + ASCVD	17,604	3.3 yrs	20% ↓ MACE

ASCVD, atherosclerotic cardiovascular disease; CVD, cardiovascular disease; MACE, major adverse cardiovascular events; T2DM, type 2 diabetes mellitus; yrs, years; ↓ indicates decrease.

**Table 3:** Direct vascular and anti-atherosclerotic mechanisms of incretin signaling.

Mechanism	GLP-1RA	GIP RA
Endothelial NO signaling [48,49]	↑ eNOS activation, ↑ NO bioavailability	limited data; indirect effects
Endothelial inflammation [50,51,52]	↓ NF-κB signaling, ↓ adhesion molecules	limited evidence
Macrophage foam cell formation [53,54,55,56]	↓ ox-LDL uptake, ↑ cholesterol efflux (ABCA1)	↓ CD36, ↓ ACAT-1 (preclinical)
Macrophage polarization [57,58]	↑ M2 anti-inflammatory phenotype	sparse data
VSMC proliferation & migration [59,60]	↓ proliferation, ↓ phenotypic switching	not established
Plaque stability [61,62]	↑ collagen content, ↓ MMP expression	preclinical stabilization signals

NO, nitric oxide; eNOS, endothelial nitric oxide synthase; NF-κB, nuclear factor-κB; VSMC, vascular smooth muscle cell; MMP, matrix metalloproteinase; ↑ indicates increase; ↓ indicates decrease.

## Data Availability

No new data were created or analyzed in this study. Data sharing is not applicable to this article.

## Abbreviations

The following abbreviations are used in this manuscript:

Abbreviation	Full Term
ACAT-1	Acyl-CoA:cholesterol acyltransferase-1
ApoE−/−	Apolipoprotein E knockout mouse
ASCVD	Atherosclerotic Cardiovascular Disease
ARR	Absolute risk reduction
BMI	Body mass index
BMSC	Bone marrow stromal cell
CABG	Coronary artery bypass grafting
cAMP	Cyclic adenosine monophosphate
CVD	Cardiovascular disease
CVOT	Cardiovascular outcome trial
DPP-IV	Dipeptidyl peptidase-IV
eNOS	Endothelial nitric oxide synthase
FDA	Food and Drug Administration
GIP	Glucose-dependent insulinotropic polypeptide
GIPR	Glucose-dependent insulinotropic polypeptide receptor
GLP-1	Glucagon-like peptide-1
GIP-RA(s)	GIP receptor agonist(s)
GLP-1RA(s)	GLP-1 receptor agonist(s)
HDL	High-density lipoprotein cholesterol
HFpEF	Heart failure with preserved ejection fraction
IRA(s)	Incretin receptor agonist(s)
LDL	Low-density lipoprotein
MACE	Major adverse cardiovascular events
MAPK	Mitogen-activated protein kinase
MI	Myocardial infarction
MMP(s)	Matrix metalloproteinase(s)
NF-κB	Nuclear factor kappa-light-chain-enhancer of activated B cells
NO	Nitric oxide
NT-proBNP	N-terminal pro–B-type natriuretic peptide
OA	Osteoarthritis
ox-LDL	Oxidized low-density lipoprotein
PKA	Protein kinase A
SBP	Systolic blood pressure
T2DM	Type 2 diabetes mellitus
TG	Triglycerides
TNF-α	Tumor necrosis factor-alpha
VLDL	Very low-density lipoprotein
VSMC(s)	Vascular smooth muscle cell(s)
